# Supplementary material for: Autotetraploid Emergence via Somatic Embryogenesis in Vitis vinifera Induces Marked Morphological Changes in Shoots, Mature Leaves, and Stomata
Source: Cells. 2021 May 28;10(6):1336. doi: 10.3390/cells10061336 (PMC8228502; doi:10.3390/cells10061336)
Supplement: Supplementary file 1 [file cells-10-01336-s001.zip › cells-1218766-supplementary.pdf]

cells MDPI

Supplementary Material

Autotetraploid Emergence via Somatic Embryogenesis in Vitis Vinifera Induces Marked Morphological Changes in Shoots, Mature Leaves and Stomata

Caterina Catalano [1,†], Loredana Abbate [1,†], Antonio Motisi [1], Dalila Crucitti [1], Vincenzo Cangelosi [2], Antonino Pisciotta [2], Rosario Di Lorenzo [2], Francesco Carimi [1,*] and Angela Carra [1]

[1] Istituto di Bioscienze e BioRisorse, Consiglio Nazionale delle Ricerche, Corso Calatafimi 414, 90129 Palermo, Italy; caterina.catalano@ibbr.cnr.it (C.C.); loredana.abbate@ibbr.cnr.it (L.A.); antonio.motisi@ibbr.cnr.it (A.M.); dalila.crucitti@ibbr.cnr.it (D.C.); angela.carra@ibbr.cnr.it (A.C.)
[2] Dipartimento di Scienze Agrarie e Forestali Università degli Studi di Palermo, Viale delle Scienze, Ed. 4, 90128 Palermo, Italy; vincenzocangelosi03@gmail.com (V.C.); antonino.pisciotta@unipa.it (A.P.); rosario.dilorenzo@unipa.it (R.D.L.)
* Correspondence: francesco.carimi@ibbr.cnr.it
† These authors have equally contributed to the study.

Table S1. RAPD, ISSR and SSR amplification profiles of *in vitro* propagated grapevine genotypes.

Molecular Marker	Marker Name	Amplicon Size Range (bp)	No. of Total Bands	Size of Each Amplicon (bp)
**RAPD**				
1.	OPAT14	200–2,000	8	200, 500, 630, 730, 1,000, 1,600, 1,700, 2,000
2.	OPH15	250–3,100	8	250, 900, 1,000, 1,500, 1,600, 2,300, 3,000, 3,100
3.	OPM04	369–1,250	7	369, 450, 550, 700, 900, 1,100, 1,250
4.	UBC219	700–3,000	5	700, 1,100, 1,700, 1,800, 3,000
5.	UBC234	700–2,700	3	700, 1,800, 2,700
6.	UBC239	600–1,850	7	600, 630, 860, 980, 1,100, 1,230, 1,850
7.	UBC247	500–2,300	7	500, 750, 850, 1,500, 1,850, 1,950, 2,300
8.	UBC251	730–2,300	5	730, 950, 1,200, 1,550, 2,300
**ISSR**				
1.	UBC855	650–2,200	6	650, 900, 1,600, 1,700, 1,950, 2,200
2.	UBC834	360–2,700	8	360, 500, 700, 800, 1,200, 1,700, 2,200, 2,700
3.	UBC851	400–1,450	9	400, 450, 600, 750, 800, 950, 1,100, 1,200, 1,450
4.	UBC841	700–2,700	6	700, 900, 1,230, 1,700, 2,200, 2,700
5.	UBC848	600–2,800	6	600, 630, 860, 1,100, 1,850, 2,800
6.	ENEA7-9	1,200–3,000	11	1,200, 1,300, 1,400, 1,500, 1,600, 1,700, 1,800, 2,000, 2,400, 2,500, 3,000
7.	ENEA12	1,000–3,500	10	1,000, 1,100, 1,200, 1,300, 1,400, 1,500, 1,900, 2,900, 3,000, 3,500
8.	ISSR2+2b	1,100–2,400	4	1,100, 1,500, 2,000, 2,400
9.	ISSR 3+3b	300–2,500	8	300, 700, 800, 900, 1,000, 2,000, 2,400, 2,500
10.	ISSR11+11b	700–3,000	7	700, 1,000, 1,100, 1,500, 1,600, 2,000, 3,000
11.	ISSR1-6	1,100–3,500	7	1,100, 1,300, 1,700, 1,900, 2,000, 3,100, 3,500
12.	ENEA21	700–2,100	9	700, 800, 1,000, 1,100, 1,300, 1,700, 1,800, 2,000, 2,100
13.	ENEA34	750–2,000	7	750, 900, 1,000, 1,100, 1,300, 1,700, 2,000
14.	ENEA36	1,100–3,500	7	1,100, 1,300, 2,000, 2,400, 2,500, 3,000, 3,500

Molecular Marker	Marker Name	Allele Size Range (bp)	Chromosome	Position (bp)	Size of Each Allele (bp)

**SSR**					
1.	VVMD7	231–268	7	1,560,528	241
2.	VVMD24	200–226	14	24,25,864	209
3.	VVMD25	234–272	11	2,971,693	242/256
4.	VVMD27	172–218	5	4,472,022	175/181
5.	VVIb01	278–318	2	2,161,587	289/295
6.	VVIh54	139–187	13	3,434,661	165/173
7.	VVIp31	158–210	19	6,697,524	185/192
8.	VVIp60	291–348	1	9,196,429	318/340
9.	VVIq52	71–89	9	21,558,043	89
